# MicroRNAs as Potential Mediators for Cigarette Smoking Induced Atherosclerosis

**DOI:** 10.3390/ijms19041097

**Published:** 2018-04-06

**Authors:** Yuka Yokoyama, Nathan Mise, Yuka Suzuki, Saeko Tada-Oikawa, Kiyora Izuoka, Lingyi Zhang, Cai Zong, Akira Takai, Yoshiji Yamada, Sahoko Ichihara

**Affiliations:** 1 Department of Human Functional Genomics, Advanced Science Research Promotion Center, Mie University, 1577 Kurimamachiya, Tsu 514-8507, Japan; ykyk49264926@gmail.com (Y.Y.); yamada@gene.mie-u.ac.jp (Y.Y.); 2 Department of Environmental and Preventive Medicine, School of Medicine, Jichi Medical University, 3311-1, Yakushiji, Shimotsuke 329-0498, Japan; nmise@jichi.ac.jp; 3 Graduate School of Regional Innovation Studies, Mie University, 1577 Kurimamachiya, Tsu 514-8507, Japan; suzujohn@yahoo.co.jp (Y.S.); t-saeko@sugiyama-u.ac.jp (S.T.-O.); Izuoka@innov.mie-u.ac.jp (K.I.); 4 Department of Occupational and Environmental Health, Tokyo University of Science, 2641 Yamazaki, Noda 278-8510, Japan; lingyiz@gmail.com (L.Z.); zongcai.nagoya@gmail.com (C.Z.); 5 Department of Physiology, Asahikawa Medical College, 2-1-1-1 Midorigaoka Higashi, Asahikawa 078-8510, Japan; takai@asahikawa-med.ac.jp

**Keywords:** cigarette smoke, atherosclerosis, NADPH oxidase, microRNAs

## Abstract

Smoking increases the risk of atherosclerosis-related events, such as myocardial infarction and ischemic stroke. Recent studies have examined the expression levels of altered microRNAs (miRNAs) in various diseases. The profiles of tissue miRNAs can be potentially used in diagnosis or prognosis. However, there are limited studies on miRNAs following exposure to cigarette smoke (CS). The present study was designed to dissect the effects and cellular/molecular mechanisms of CS-induced atherosclerogenesis. Apolipoprotein E knockout (ApoE KO) mice were exposed to CS for five days a week for two months at low (two puffs/min for 40 min/day) or high dose (two puffs/min for 120 min/day). We measured the area of atherosclerotic plaques in the aorta, representing the expression of miRNAs after the exposure period. Two-month exposure to the high dose of CS significantly increased the plaque area in aortic arch, and significantly upregulated the expression of atherosclerotic markers (*VCAM-1*, *ICAM-1*, *MCP1*, *p22phox*, and *gp91phox*). Exposure to the high dose of CS also significantly upregulated the miRNA-155 level in the aortic tissues of ApoE KO mice. Moreover, the expression level of miR-126 tended to be downregulated and that of miR-21 tended to be upregulated in ApoE KO mice exposed to the high dose of CS, albeit statistically insignificant. The results suggest that CS induces atherosclerosis through increased vascular inflammation and NADPH oxidase expression and also emphasize the importance of miRNAs in the pathogenesis of CS-induced atherosclerosis. Our findings provide evidence for miRNAs as potential mediators of inflammation and atherosclerosis induced by CS.

## 1. Introduction

Atherosclerosis is the leading cause of death in the developed world [1]. It is a chronic disease in which systemic inflammation underlies the accumulation of plaques in the arterial intima. Atherosclerotic plaques can obstruct the arterial lumen, leading to cardiovascular diseases (CVD), such as coronary artery disease, myocardial infarction, heart attack, or ischemic stroke [2]. The mechanisms underlying atherosclerosis have been analyzed extensively in clinical studies, animal experiments, and in vitro preparations [2,3,4]. Epidemiological studies have shown that cigarette smoking, hypercholesterolemia, hypertension, and diabetes are the major risk factors for progression of atherosclerosis and related diseases [4]. Among them, cigarette smoking (CS) is an important risk factor for the progression of atherosclerosis and CVD [5,6,7]. Exposure to CS enhances virtually all aspects of atherogenesis, including oxidative stress responses, endothelial dysfunction and activation of macrophages, and lipid disposition of foam cells [7,8,9].

The apolipoprotein E knockout (ApoE KO) mouse is the most widely used animal model for human CVD [10]. The lack of the *Apoe* gene in ApoE KO mice causes delay of lipoprotein clearance and results in hyper- and dys-lipoproteinemia, hypercholesterolemia, and atherosclerotic lesions, even under a normal diet condition [11]. The ApoE KO mouse shows all phases of lesions observed in human atherogenesis [12]. Previous studies demonstrated accelerated atherosclerosis in ApoE KO mice exposed to CS [12,13].

Recent studies have suggested that non-coding RNAs (ncRNAs) possessing no or little protein coding potential have important functions in various biological processes [14]. Among the ncRNAs, microRNAs (miRNAs) are the best-known classes of endogenous ncRNAs that are approximately 22-nucleotides in length and control the expression of messenger RNAs (mRNAs) at the posttranscriptional level. The miRNA regulation of molecular pathways correlates with virtually all stages of CVD development [15,16,17]. Furthermore, differential expression of miRNAs is observed between disease and normal tissues. Global expression profiling of miRNAs in cells and tissues exposed to CS confirmed their involvement in the CVD process and disease. Noninvasive sampling of miRNAs is possible from blood, saliva, and urine samples [15,18], thus, the expression patterns of miRNAs can be considered as promising biomarkers.

Altered expression of miRNAs has been reported in various diseases and the profiles of tissue miRNAs exhibit potential applications in diagnosis and prognosis [19,20]. While the role of ncRNAs and in particular that of miRNAs is becoming increasingly clear in diseases like cancer [21,22], such a role of miRNAs is only partially clarified in CVD. Previous studies have revealed the importance of miRNAs in the regulation of lipid homeostasis, endothelial cell inflammation, leukocyte recruitment, and vascular smooth muscle function [19]. However, there are limited studies on miRNAs during CS-enhanced atherosclerogenesis. The present study was designed to dissect the effects and cellular/molecular mechanisms of CS-induced atherosclerogenesis in ApoE KO. Furthermore, we focused on the miRNAs in regulation of endothelial cell inflammation, especially regulation of adhesion molecules that alter the balance of atherosclerotic plaque progression and regression. We screened the expression of those miRNAs to identify the differentially expressed miRNAs in the aortic tissues of mice exposed to CS.

## 2. Results

### 2.1. Changes in Body, Lung, Liver, and Kidney Weights

ApoE KO mice were exposed to CS at low (two puffs/min for 40 min/day) or high dose (two puffs/min for 120 min/day) for five days a week for two months. After two months, body weight was significantly lower in ApoE KO mice exposed to CS at a high dose than the control group (27.6 ± 0.6 vs. 30.9 ± 0.3 g, mean ± SEM) (Figure S1a). There was no significant difference in body weight between the ApoE KO mice exposed to CS at a low dose and the control group. Furthermore, exposure to CS tended to lower lung, liver, and kidney weights, irrespective of the dose, but the weight reduction was not statistically significant (Figure S1b–d).

### 2.2. Effects of CS on Atherosclerosis in ApoE KO Mice

**Figure 1:**
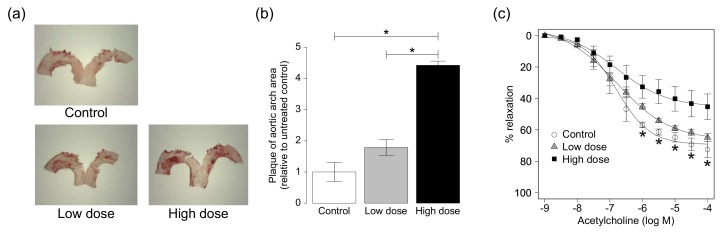
Plaque formation in aortic arch of ApoE KO mice. (**a**) Representative images of aortic arch stained with oil red-O solution and (**b**) plaque area in aortic arch of ApoE KO mice exposed to CS. Quantitative data are expressed relative to the values of the control group. Data are mean ± SEM of six animals per group; (**c**) Cumulative acetylcholine-induced relaxation in aortic rings from ApoE KO mice exposed to CS. Data are mean ± SEM of four-six animals per group. * *p* < 0.05 versus the high-dose group.

The percentage of the plaque area was determined by staining of the aortic arch with oil red-O solution. The extent of atherosclerosis was significantly larger in the aortic arch of ApoE KO mice exposed to the high dose of CS than the control and the low dose (Figure 1a,b). There was no significant difference between ApoE KO mice exposed to the low dose of CS and the control group.

### 2.3. Effects of High-Dose CS on Acetylcholine-Induced Vasorelaxation

In vitro relaxation studies were conducted using endothelium-intact aortic rings exposed to 10^−7^ M phenylephrine. Acetylcholine induced cumulative concentration-dependent relaxation in phenylephrine precontracted aortic rings. The acetylcholine-induced relaxation curve shifted upward and a decrease in relaxation was observed in the aortas of ApoE KO mice exposed to CS at the high dose (Figure 1c).

### 2.4. Effects of High-Dose CS on VCAM-1, ICAM-1, and MCP1 Expression in Thoracic Aorta

**Figure 2:**
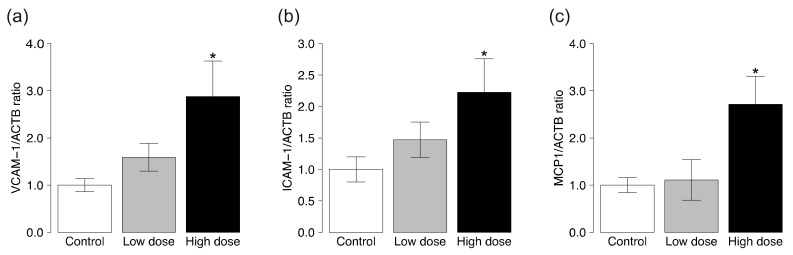
Gene expression levels of *VCAM-1*, *ICAM-1*, and *MCP1* in ApoE KO mice exposed to CS. The mRNA levels of (**a**) *VCAM-1*; (**b**) *ICAM-1*; and (**c**) *MCP1* in the aortic tissues were determined by quantitative RT-PCR analysis. Data are normalized by the abundance of *β-actin* mRNA. Quantitative data are expressed relative to the values of the control group. Data are mean ± SEM of seven animals per group. * *p* < 0.05 versus the control group.

Next, we tested the effects of CS on the expression of *vascular cell adhesion molecule-1* (*VCAM-1*), *intercellular adhesion molecule-1* (*ICAM-1*), and *monocyte chemotactic protein 1* (*MCP1*). The expression levels of *VCAM-1* and *ICAM-1* mRNA were significantly higher in the thoracic aortas of ApoE KO mice exposed to high-dose, but not low-dose, CS compared to the control group (Figure 2a,b). Likewise, the expression level of *MCP1* mRNA was higher in ApoE KO mice exposed to CS at the high dose, but not to low-dose CS, compared to the control group (Figure 2c).

### 2.5. High-Dose CS Increases Oxidative Stress Markers

**Figure 3:**
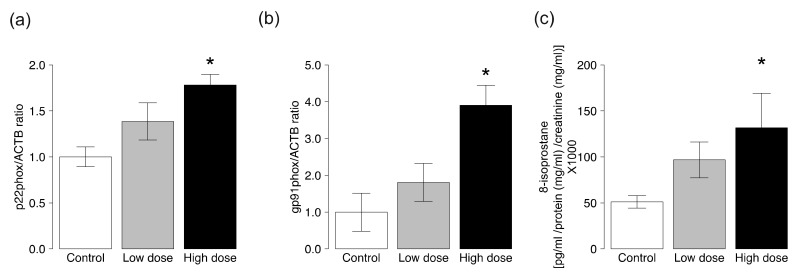
Gene expression levels of NADPH oxidase subunits and the level of 8-iso-prostaglandin F_2α_ in ApoE KO mice exposed to CS. The mRNA levels of (**a**) *p22phox* and (**b**) *gp91phox* in the aortic tissues were determined by quantitative RT-PCR analysis. Data are normalized by the abundance of *β-actin* mRNA. Quantitative data are expressed relative to the values for the control group; (**c**) 24-h urinary 8-iso-prostaglandin F_2α_ was measured using the enzyme immunoassay kit. Data are mean ± SEM of six or seven animals per group. * *p* < 0.05 versus the control group.

Since NADPH oxidase is a major source of reactive oxygen species (ROS) in cardiovascular cells including vascular smooth muscle cells and endothelial cells, we measured the expression levels of components of the membrane-associated enzyme phagocyte NADPH oxidase. The abundance of *p22phox* and *gp91phox* mRNAs in the thoracic aortas was significantly greater in ApoE KO mice exposed to high-dose CS than the control group (Figure 3a,b). Exposure to high-dose CS increased the expression levels of *p47phox* and *p67phox* mRNAs, but there were no statistically significant differences in the expression levels of these mRNAs between the groups (Figure S2a,b). Exposure to low-dose CS did not affect the expression levels of all examined mRNAs.

8-iso-prostaglandin F_2α_ is a marker of oxidative stress [23]. The levels of urinary 8-isoprostane were significantly higher in ApoE KO mice exposed to high-dose CS, but not low-dose CS, compared to the control group (Figure 3c).

### 2.6. High-Dose CS Increases Expression of miRNAs

**Figure 4:**
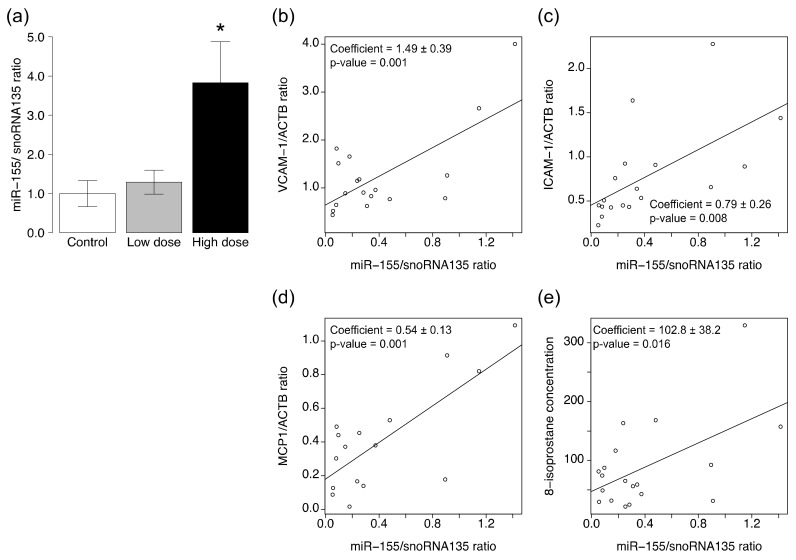
Expression levels of miRNAs in ApoE KO mice exposed to CS. The levels of (**a**) miR-155 in the aortic tissues were determined by quantitative RT-PCR analysis. Data are normalized by the abundance of snoRNA135. Quantitative data are expressed relative to the values for the control group. Data are mean ± SEM of six or seven animals per group. * *p* < 0.05 versus the control group. The scatter plots showing the correlation between expression levels of miR-155 and (**b**) *VCAM-1*; (**c**) *ICAM-1*; (**d**) *MCP1*; and (**e**) creatinine adjusted level of 24-h urinary 8-iso-prostaglandin F_2α_. The coefficients and p-values were shown in the plots.

The expression level of miR-155 was significantly higher in the aortic tissues of ApoE KO mice exposed to high-dose CS compared with the control group (Figure 4a). The expression level of miR-126 tended to be lower (Figure S3a) and that of miR-21 tended to be higher in ApoE KO mice exposed to high-dose CS than the control group (Figure S4a), albeit statistically insignificant. Exposure to low-dose CS did not affect the expression level of three miRNAs in the aortas of ApoE KO mice. Regression analyses showed significant correlations between miR-155 level and VCAM-1/ACTB ratio (*p* = 0.001, *r* = 0.69), ICAM-1/ACTB ratio (*p* = 0.008, *r* = 0.61), MCP1/ACTB ratio (*p* = 0.007, *r* = 0.75), and the levels of 8-isoprostane (*p* = 0.016, *r* = 0.55) (Figure 4b–e, Table 1). Similarly, miR-21 levels correlated with the VCAM-1/ACTB ratio (*p* = 0.004, *r* = 0.63) and MCP1/ACTB ratio (*p* = 0.006, *r* = 0.63) (Figure S4b,d, Table 1).

## 3. Discussion

In the present study, we showed that two-month exposure to high-dose CS significantly increased the plaque size in the aortic arch of ApoE KO mice, and significantly upregulated the expression of various markers of atherosclerosis, such as *VCAM-1*, *ICAM-1*, *MCP1*, *p22phox*, and *gp91phox* in mice, indicating that exposure to high-dose CS aggravated the atherosclerotic process. Our results also showed upregulation of miRNA-155 and miRNA-21 and downregulation of miRNA-126 in the aortic tissues of ApoE KO mice exposed to high-dose CS.

It has been well documented that exposure to CS can lead to the development of diseases of a variety of organ systems, especially the cardiovascular system. The association between endothelial cell injury/inflammation and the development of atherosclerosis is also well known [24]. Injury of endothelial cells promotes the migration of mononuclear cells and triggers the attachment of leukocytes to the subendothelial region, initiating the process of atherosclerogenesis. Other studies showed that CS extracts induced pro-inflammatory/matrix remodeling genes (MCP1, MMPs (matrix metalloproteinases), TNF (tumor necrosis factor)-α, IL (interleukin)-1β, NF (nuclear factor)–κB) in cultured cells [25], suggesting that the harmful effects of CS on atherosclerosis are mediated through the pro-inflammatory/matrix pathway. Our results showed upregulation of genes associated with inflammation and adhesion responses, such as *MCP1*, *VCAM-1*, and *ICAM-1*, in the aortas of ApoE KO mice exposed to CS. These results are consistent with previous studies that showed a CS-induced increase in MCP1 expression in lung tissues and bronchoalveolar lavage fluid [26,27]. Other studies showed that exposure to CS affected monocyte behavior and interaction with the endothelium [28] and demonstrated that CS enhanced endothelial cell adhesion, inflammation, and cell stress [29].

Oxidative stress plays an important role in the pathogenesis of cardiovascular diseases, such as hypertension and atherosclerosis [30]. Oxidative stress results from an imbalance between radical-generating and radical-scavenging systems, leading to cell membrane impairment. NADPH oxidase plays a pivotal role in modulating endothelial function [31]. 8-epi-prostaglandin F_2α_ is produced by free radical-dependent peroxidation of lipid-esterified arachidonic acid and is used as a biomarker of lipid peroxidation [23]. CS was reported to induce inflammation and oxidative stress in brains and lungs [32,33]. Consistent with previous studies, our results demonstrated that high-dose CS increased oxidative stress in the aortas, which likely explains the mechanisms responsible for endothelial dysfunction and accelerated atherosclerogenesis induced by CS. In the present study, we could not measure NADPH oxidase activity and the phosphorylation of NADPH complex proteins in the aortic tissues, as well as oxidative stress markers in the serum, due to the lack of sufficient samples. We need further studies to confirm the roles of ROS in CS-induced athrosclerogenesis.

The microRNA pathways control various biological processes, such as development, cellular differentiation, and pathogenesis by posttranscriptional regulation of gene expression. Several miRNAs are involved in the development of atherosclerosis [19]. In the present study, we found upregulation of VCAM-1, ICAM-1, and MCP1 in aortic tissues with atherosclerotic plaques of high-dose CS exposed ApoE KO mice. MCP1 is one of the key chemokines that regulate the migration and infiltration of monocytes/macrophages [34], and VCAM-1 and ICAM-1 are adhesion molecules that mediate the firm adhesion of leukocytes to the endothelial cells and play a critical role in subsequent leukocyte transmigration [35]. Both these genes are known to be regulated by microRNA pathways. Peroxisome proliferators-activated receptor (PPAR)-α is the target of miR-155 and -21, and downregulation of PPAR-α results in upregulation of VCAM-1, ICAM-1, and MCP1 through the activation of transcription factor AP-1 [36,37]. It has also been reported that MCP1 expression induces miR-21 biogenesis, thus forming a positive feedback loop [36]. The finding of increased expression levels of miR-155 and -21 in high-dose CS-exposed ApoE KO mice was consistent with that of previous studies reporting the correlation between VCAM-1, ICAM-1, and MCP1 proteins and miR-155 and -21 [34,35,36,38]. Upregulation of miR-155 can promote several pro-inflammatory processes, including leukocyte adhesion to the endothelium by enhancing the expression of VCAM-1 and ICAM-1, leading to atherosclerosis [39].

In addition, it has been reported that oxidative stress can induce miR-21 expression and modulate the PI3K/Akt/eNOS pathway in endothelial cells [40]. MiR-155 also plays an important role in the control of ROS and nitric oxide production by regulating the EGFR/ERK/p38 MAPK and PI3K/Akt pathways in endothelial cells [41]. The present study showed that CS exposure significantly upregulated phagocytic oxidase components, *p22phox* and *gp91phox*, as well as 8-iso-prostaglandin F_2α_, indicating that CS increases oxidative stress. These results suggest that the microRNA pathways modulate CS-induced changes in oxidative stress. 

Contrary to miR-155 and -21, miR-126 tends to be downregulated in aortic tissues in ApoE KO mice exposed to CS (Figure S3a). Previous studies showed that miR-126 directly suppressed the expression of VCAM-1 [42] and reduced oxidative stress through the upregulation of sirtuin 1 (SIRT1) and superoxide dismutase-2 (SOD-2) [43]. In addition, it has been reported that the overexpression of miR-126 downregulates MCP1 [44]. Although a statistically significant difference was not observed, the upregulation of VCAM-1 and MCP1 and downregulation of miR-126 observed in the present study are consistent with the results of the abovementioned studies. Considered together, the above findings and the present study support the possibility that miR-126 protects against the development of atherosclerosis. Furthermore, the above results suggest that downregulation of atheroprotective miRNAs, such as miR-126, induced by exposure to CS, increased the expression of the phagocytic oxidase complex. However, we need further studies since no significant changes were observed in the expression level of miR-126 after exposure to CS. Consistent with the present study, previous studies have shown that the miR-126 level was decreased in the aortas of ApoE KO mice fed a high-fat diet [45]. Conversely, other previous studies have shown that the miR-126 level was increased in the aortas of ApoE KO mice with superimposed chronic kidney disease [46]. Fish et al. have shown that miR-126 regulates many aspects of endothelial cell biology, including cell migration, organization of the cytoskeleton, capillary network stability, and cell survival [47]. The expression levels of miR-126 might be different depending on the pathophysiological conditions. Further studies are needed to clarify the association between the pathophysiological conditions and the regulation mechanisms of miRNAs.

In the human studies, it has been described that the miR-155 level was significantly upregulated in the plasma of coronary artery diseases patients [48] and was elevated in the plasma and plaque of patients with atherosclerosis [49]. Moreover, the miR-21 level was upregulated in plasma of patients with acute myocardial infarction [50]. The present study highlighted the crucial role of miRNAs in CS-induced atherosclerosis and the association between the expression of the adhesion molecules and miRNA levels in the animal model. Studies underling the significance of plasma miRNAs in the diagnosis of diseases related to atherosclerosis should be continued in order to establish them as a potential biomarker for such diseases.

## 4. Materials and Methods

### 4.1. Animal Experiments

B6.129P2-Apoe^tm1Unc^ (ApoE^−/−^) mice were obtained from Jackson Laboratory (Bar Harbor, ME, USA). To evaluate the effects of CS on atherosclerogenesis, ApoE KO mice were exposed to CS at low (two puffs/min for 40 min/day) or high dose (two puffs/min for 120 min/day) (*n* = 7, each group) for five days a week for two months. The CS inhalation were conducted using unfiltered research cigarette 3R4F (Tabacco Health Research Institute, Kentucky University, Lexington, KY, USA) and the Tabacco Smoke Inhalation Experiment System for small animals (Model SIS-CS; Shibata Scientific Technology, Tokyo, Japan) [51]. The smoke generator automatically generated CS by setting the volume of the syringe pump and the number of puffs per minutes. The generated CS was delivered to the inhalation chamber to which the mice body holders were set and mice were exposed to CS through the nose. Cigarettes were smoked according to a modified Federal Trade Commission protocol at a rate of two puffs/min, two s/puff, and 35 mL/puff [52]. The animals were housed in a temperature- and light-controlled environment at 25 °C with a 12-h light–dark cycle. Body weight was measured each week. The experimental protocol was approved by the Animal Research Committee of Mie University and all animal procedures were conducted humanely in accordance with the guidelines for the care and use of laboratory animals approved by our university (Approval No. 24-29-1, 29 October 2015).

### 4.2. Quantitative Assessment of Atherosclerosis

After two months of CS exposure, each body organ was carefully dissected out after sacrifice and weighed. The aortic arch was harvested and fixed in PBS with 4% paraformaldehyde and the adventitia was removed under a microscope, as described in detail previously [53]. Then, the aortic arch was opened longitudinally, immersed for 1 min in 60% isopropanol, and stained with oil red-O solution for 15 min at 37 °C. All images were captured with a microscope equipped with a camera (EZ4HD, Leica, Wetzlar, Germany) and analyzed using Image J software (NIH, Bethesda, MD, USA). The edge of the aorta was traced using an automated feature and the extent of atherosclerosis was determined by selecting threshold ranges in the three basic colors of Image J software. The total aortic surface area and the lesion area were then calculated. The extent of atherosclerosis was expressed as the percent of surface area of the aorta covered by the lesions [54].

### 4.3. Measurement of Vascular Reactivity of Thoracic Aortic Rings

The thoracic aorta was rapidly removed after the chest was opened. The aortas were then placed in Krebs-Henseleit (K-H) solution [in mmol/L: NaCl 119.0, KCl 4.7, CaCl_2_ 2.5, KH_2_PO_4_ 1.2, MgSO_4_ 1.2, NaHCO_3_ 25.0, and glucose 11.0 (pH 7.4)] [55]. After removal of the superficial connective tissue, the aortas were cut into 3-mm thick rings and mounted in 5.0 mL organ baths containing the K-H solution. The bath solution was maintained at 37 °C and bubbled continuously with a mixture of 95% O_2_ and 5% CO_2_. The aortic rings were equilibrated for 60 min at a resting tension of 1 g. The contractile response of the aortic ring was measured using a force transducer (East Magnus System; Iwashiya Kishimoto, Kyoto, Japan) [56]. Phenylephrine at a final concentration of 10^−7^ M was added to the bath to contract the ring and force was allowed to stabilize for 15 min. Afterwards, vasorelaxation was tested with acetylcholine (from 10^−9^ to 10^−4^ M) [57].

### 4.4. RNA Isolation and Analysis of RT-PCR

The mRNA expression levels of *VCAM-1*, *ICAM-1*, and *MCP1* were determined in the aortas by RT-PCR. About 2 mg of frozen aortic tissue was homogenized, and total RNA was isolated using the ReliaPrep^TM^ RNA Tissue Miniprep System (Promega, Madison, WI, USA) according to the protocol provided by the manufacturer. The concentration of total RNA was quantified by spectrophotometry (ND-1000; NanoDrop Technologies, Wilmington, DE, USA). The A260/A280 ratio of the spectrograms was confirmed between 1.8–2.0, and the A260/A230 ratio was always greater than 1.5. RNA was reverse transcribed to single-stranded cDNA using the SuperScript III First-Strand Synthesis System for RT-PCR (Thermo Fisher Scientific, Waltham, MA, USA). The cDNA was subjected to quantitative PCR analysis with FastStart Universal Probe Master Mix (Roche, Basel, Switzerland) using primers for *VCAM-1*, *ICAM-1*, and *MCP-1* using an ABI 7300 Real-Time PCR system (Thermo Fisher Scientific), as described previously [58]. The primers and probes were designed by the Universal ProbeLibrary Assay Design Center (Roche, Basel, Switzerland). The relative level of mRNA expression of each gene was expressed relative to that of *β-actin*.

### 4.5. Analysis of Markers of Oxidative Stress

We tested the effects of CS on the expression of NADPH oxidase subunits. The mRNA expression levels of *p22phox*, *p47phox*, *p67phox*, and *gp91phox* in aortas were determined by RT-PCR. cDNA was subjected to quantitative PCR analysis with FastStart Universal Probe Master Mix (Roche, Basel, Switzerland) using primers for each gene and the ABI 7300 Real-Time PCR system (Thermo Fisher Scientific). The primers and probes were designed by the Universal ProbeLibrary Assay Design Center (Roche, Basel, Switzerland). The relative mRNA expression level of each gene was normalized to that of β-actin.

Twenty-four-hour urine was collected using metabolic cages (Natsume Seisakusho, Tokyo, Japan) on the last day of exposure to CS. To evaluate oxidative stress, we measured 24-h urinary 8-iso-prostaglandin F_2α_ using the enzyme immunoassay kit (Cayman Chemical, Ann Arbor, MI, USA). Urine samples were purified using 8-Isoprostane Affinity Purification Kit (Cayman Chemical) before measurement and urinary creatinine concentrations were obtained by the colorimetric method (Abcam, Cambridge, UK).

### 4.6. Analysis of miRNA Expression Levels

About 2 mg of frozen aortic tissue was homogenized, and total RNA including small RNAs was isolated using the mirVana miRNA Isolation Kit (Thermo Fisher Scientific) according to the protocol provided by the manufacturer. The concentration of total RNA was quantified by spectrophotometry (ND-1000; NanoDrop Technologies, Wilmington, DE, USA). Single-stranded cDNA was synthesized using the TaqMan MicroRNA Reverse Transcription Kit (Thermo Fisher Scientific) and subjected to quantitative PCR analysis with TaqMan Universal master Mix II (Thermo Fisher Scientific) using TaqMan MicroRNA Assays (Thermo Fisher Scientific) for miRNA-155 (mmu-miR-155-5p), miRNA-126 (mmu-miR-126a-3p), and miR-21 (mmu-miR-21a-5p), as described in detail previously [59]. Several transfer RNA (tRNA), small nuclear RNA (snRNA), and small nucleolar RNA (snoRNA) were used as the endogenous controls for real-time quantitation of microRNAs. Mouse snoRNA135 was used as the control in the present study, as it was successful in previous studies measuring the levels of microRNA associated with exposure to cigarette smoke [60,61]. The relative levels of miRNA expression were normalized to that of snoRNA135.

### 4.7. Statistical Analysis

All parameters were expressed as mean ± standard error of the mean (SEM). Statistical analyses were performed using one-way analysis of variance (ANOVA) followed by Dunnett’s post hoc test. Regression analyses were performed between the VCAM-1/ACTB ratio, ICAM-1/ACTB ratio, MCP1/ACTB ratio, and the level of 8-epi-prostaglandin F_2α_ for each miRNA level. A *p* value less than 0.05 was considered statistically significant. All statistical analyses were performed using the JMP 8.0 software (SAS Institute, Cary, NC, USA).

## 5. Conclusions

Exposure to CS induces changes in not only atherosclerosis marker proteins, but also microRNAs profiles. The present study demonstrated that exposure to high-dose CS induced atherosclerosis through the upregulation of miR-155 in aortas of ApoE KO mice. Moreover, the expression level of miR-126 tended to be downregulated and that of miR-21 tended to be upregulated in ApoE KO mice exposed to high-dose CS compared with the control group, albeit statistically insignificant. The results suggest that CS induces atherosclerosis through increased vascular inflammation and NADPH oxidase expression and also emphasize the importance of miRNAs in the pathogenesis of CS-induced atherosclerosis. Our findings provide evidence for miRNAs as potential mediators of inflammation and atherosclerosis induced by CS.

## Figures and Tables

**Table 1:** The coefficient and *p*-value of the regression line analysis.

miRNAs	VCAM-1/ACTB		ICAM-1/ACTB		MCP1/ACTB		8-Isoprostane	
	Coefficient	*p*-Value	Coefficient	*p*-Value	Coefficient	*p*-Value	Coefficient	*p*-Value
miR-155	1.49 ± 0.39	0.001	0.79 ± 0.26	0.008	0.54 ± 0.13	0.007	102.8 ± 38.2	0.016
miR-126	−0.50 ± 0.63	0.435	−0.48 ± 0.36	0.204	−0.14 ± 0.22	0.534	−45.2 ± 51.4	0.391
miR-21	17.82 ± 5.26	0.004	6.71 ± 3.73	0.089	6.07 ± 1.92	0.006	670.4 ± 471.1	0.172

Data are mean ± SEM.

## Supplementary Materials

Supplementary materials can be found at http://www.mdpi.com/1422-0067/19/4/1097/s1.

## Abbreviations

VCAM-1	vascular cell adhesion molecule-1
ICAM-1	intercellular adhesion molecule-1
MCP1	monocyte chemotactic protein 1
ACTB	actin, beta
SEM	standard error of the mean
