# Supplementary material for: MicroRNAs as Potential Mediators for Cigarette Smoking Induced Atherosclerosis
Source: Int J Mol Sci. 2018 Apr 6;19(4):1097. doi: 10.3390/ijms19041097 (PMC5979571; doi:10.3390/ijms19041097)
Supplement: Supplementary file 1 [file ijms-19-01097-s001.pdf]

Supplementary

MicroRNAs as Potential Mediators for Cigarette Smoking Induced Atherosclerosis

Yuka Yokoyama [1], Nathan Mise [2], Yuka Suzuki [3], Saeko Tada-Oikawa [3], Kiyora Izuoka [3], Lingyi Zhang [4], Cai Zong [4], Akira Takai [5], Yoshiji Yamada [1] and Sahoko Ichihara [1,2,3,*]

1 Department of Human Functional Genomics, Advanced Science Research Promotion Center, Mie University, 1577 Kurimamachiya, Tsu 514-8507, Japan; ykyk49264926@gmail.com (Y.Y.); yamada@gene.mie-u.ac.jp (Y.Y.)
2 Department of Environmental and Preventive Medicine, Jichi Medical University School of Medicine, 3311-1, Yakushiji, Shimotsuke 329-0498, Japan; nmise@jichi.ac.jp (N.M.)
3 Graduate School of Regional Innovation Studies, Mie University, 1577 Kurimamachiya, Tsu 514-8507, Japan; suzujohn@yahoo.co.jp (Y.S.); t-saeko@sugiyama-u.ac.jp (S.T.-O.); Izuoka@innov.mie-u.ac.jp (K.I.)
4 Department of Occupational and Environmental Health, Tokyo University of Science, 2641 Yamazaki, Noda 278-8510, Japan; lingyiz@gmail.com (L.Z.); zongcai.nagoya@gmail.com (C.Z.)
5 Department of Physiology, Asahikawa Medical College, 2-1-1-1 Midorigaoka Higashi, Asahikawa 078-8510, Japan; takai@asahikawa-med.ac.jp (A.T.)

* Correspondence: saho@jichi.ac.jp; Tel.: +81-285-58-7335; Fax: +81-285-44-8645

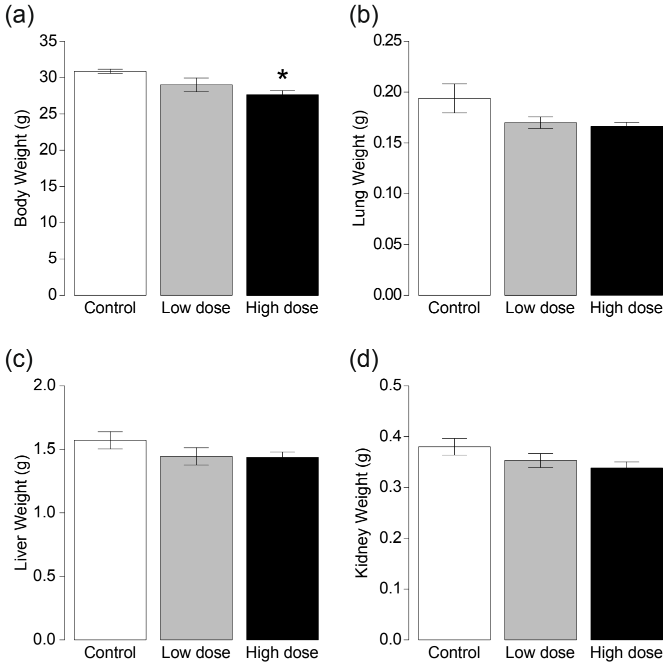

Figure S1. Changes in (a) body, (b) lung, (c) liver, and (d) kidney weights of ApoE KO mice after 2-month exposure to CS at low or high dose. Data are mean±SEM of seven animals per group. **P*<0.05 versus the control group.

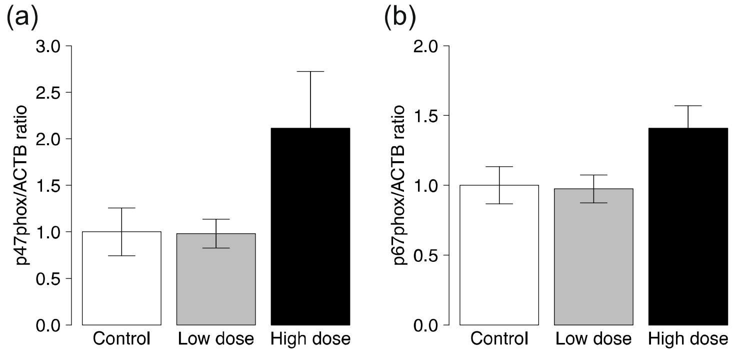

Figure S2. Gene expression levels of NADPH oxidase subunits in ApoE KO mice exposed to CS. The mRNA levels of (a) *p47phox* and (b) *p67phox* in the aortic tissues were determined by quantitative RT-PCR analysis. Data are normalized by the abundance of *β-actin* mRNA. Quantitative data are expressed relative to the values for the control group. Data are mean±SEM of six or seven animals per group.

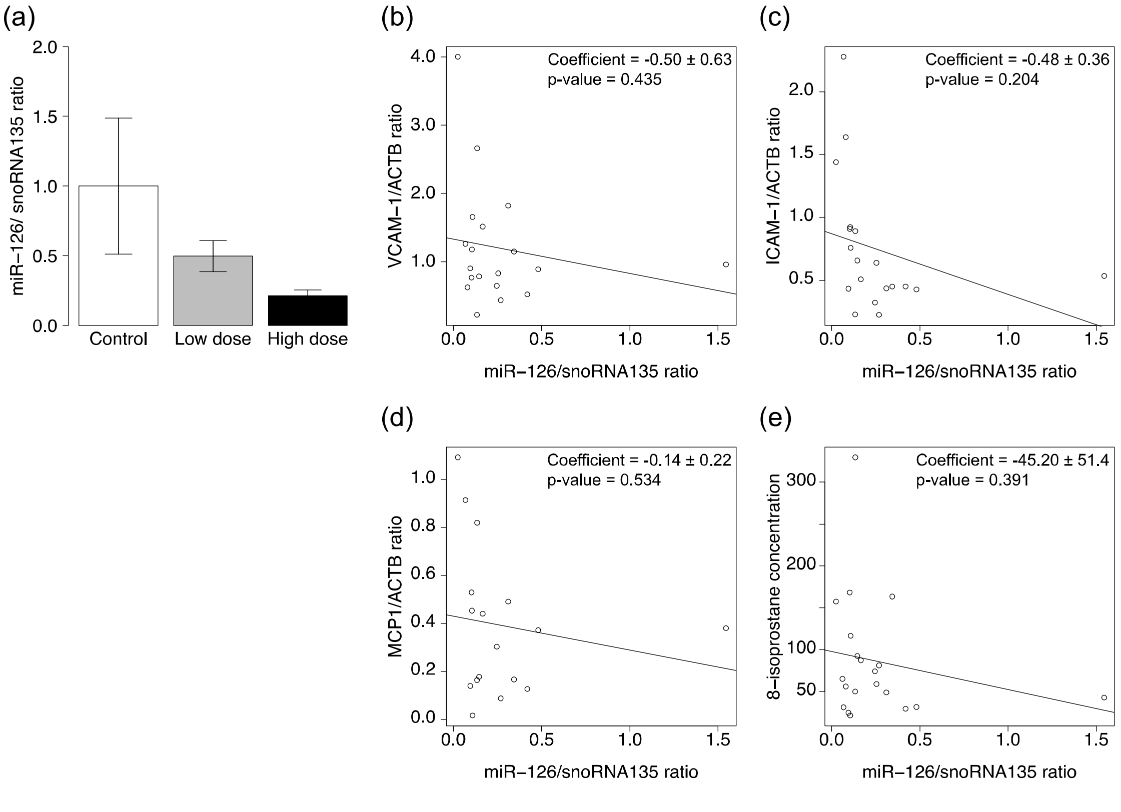

Figure S3. Expression levels of miRNAs in ApoE KO mice exposed to CS. The levels of (a) miR-126 in the aortic tissues were determined by quantitative RT-PCR analysis. Data are normalized by the abundance of snoRNA135. Quantitative data are expressed relative to the values for the control group. Data are mean±SEM of six or seven animals per group. The scatter plots showing the correlation between expression levels of miR-126 and (b) *VCAM-1*, (c) *ICAM-1*, (d) *MCP1*, and (e) creatinine adjusted level of 24-h urinary 8-iso-prostaglandin $F_{2\alpha}$. The coefficients and *p*-values were shown in the plots.

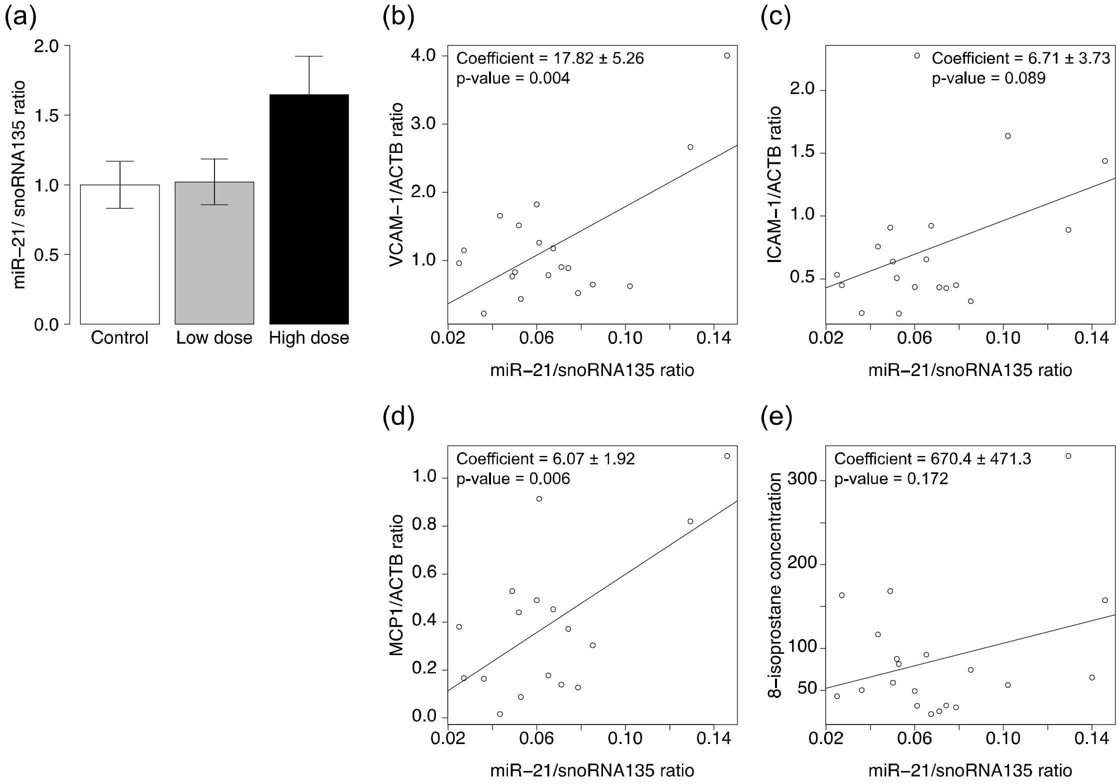

Figure S4. Expression levels of miRNAs in ApoE KO mice exposed to CS. The levels of (a) miR-21 in the aortic tissues were determined by quantitative RT-PCR analysis. Data are normalized by the abundance of snoRNA135. Quantitative data are expressed relative to the values for the control group. Data are mean±SEM of six or seven animals per group. The scatter plots showing the correlation between expression levels of miR-21 and (b) *VCAM-1*, (c) *ICAM-1*, (d) *MCP1*, and (e) creatinine adjusted level of 24-h urinary 8-iso-prostaglandin $F_{2\alpha}$. The coefficients and p-values were shown in the plots.
